# Comparative Evaluation of Different Biomass Ashes as Supplementary Cementitious Materials: Reactivity, Hydration Impact and Environmental Considerations

**DOI:** 10.3390/ma18184239

**Published:** 2025-09-09

**Authors:** Steffen Overmann, Isabell Allwicher, David Montag, Anya Vollpracht, Thomas Matschei

**Affiliations:** 1 Institute of Building Materials Research (ibac), RWTH Aachen University, Schinkelstr. 3, 52062 Aachen, Germany; vollpracht@ibac.rwth-aachen.de (A.V.); matschei@ibac.rwth-aachen.de (T.M.); 2 Institute of Environmental Engineering (ISA), RWTH Aachen University, Mies-van-der-Rohe-Str. 1, 52074 Aachen, Germany; allwicher@isa.rwth-aachen.de (I.A.); montag@isa.rwth-aachen.de (D.M.)

**Keywords:** wood ash, spelt husk ash, sewage sludge ash, phosphorus recovery, supplementary cementitious material, cement

## Abstract

Biomass ashes are considered to be sustainable alternatives for fly ashes from hard coal combustion for the use as supplementary cementitious material (SCM). However, their diverse composition and properties are impeding their standardized use. This study aims to gain a better understanding of how composition affects performance. It investigates three wood ashes (one bottom ash, two fly ashes), one spelt husk ash and a mineral residue from sewage sludge ash after wet-chemical phosphorus recovery for their suitability as SCM. After characterization of the materials including the determination of environmentally relevant parameters, the reactivity was tested using the R^3^ test and mortar compressive strength with different substitution levels. The effect on hydration was studied in blends with Portland cement using isothermal calorimetry and X-ray diffractometry (XRD). The composition of the ashes differed significantly, also between the wood ashes. The wood ashes showed no significant reactivity (cumulative R^3^ heat lower than 125 J/g SCM after 7 days), while the spelt husk ash and the sewage sludge ash residue showed distinct reactivity with a cumulative R^3^ heat of 249 and 181 J/g SCM after 7 days, respectively. Following an initial period of unaffected hydration, the wood fly ashes were found to impede clinker reactivity. In contrast, the other materials exhibited no significant influence on the hydration process, aside from the consumption of portlandite by the reactive ones. The wood fly ashes also impaired strength development in blended mortar formulations (e.g., relative compressive strengths with a cement substitution level of 20 wt% after 28 days were <0.6), whereas the reactive spelt husk ash and the mineral residue were associated with a measurable contribution to strength gain (e.g., relative compressive strengths with a cement substitution level of 20 wt% after 28 days were >0.85). The wood bottom ash was the only material investigated which perfectly sustained mortar workability and rather acts like a nearly inert addition. The results show both the potential and the limitations of using different types of ash, which cannot be generalized due to the wide variation in raw materials and combustion conditions.

## 1. Introduction

According to estimates, the cement industry is responsible for about 8% of global CO_2_ emissions caused by the clinker production [1]. Because of the ongoing anthropogenic climate change, there are great efforts to reduce the proportion of clinker in cement by using alternative materials, so-called supplementary cementitious materials (SCMs). Due to process changes in the German steel industry and closures of coal-fired power plants, the availability of the most used conventional SCMs, blast furnace slag and fly ash, will decrease in the near future [2]. In conjunction with the existing climate policy targets, it is foreseeable that the expected increase in demand cannot be met without novel SCMs.

**Figure 1:**
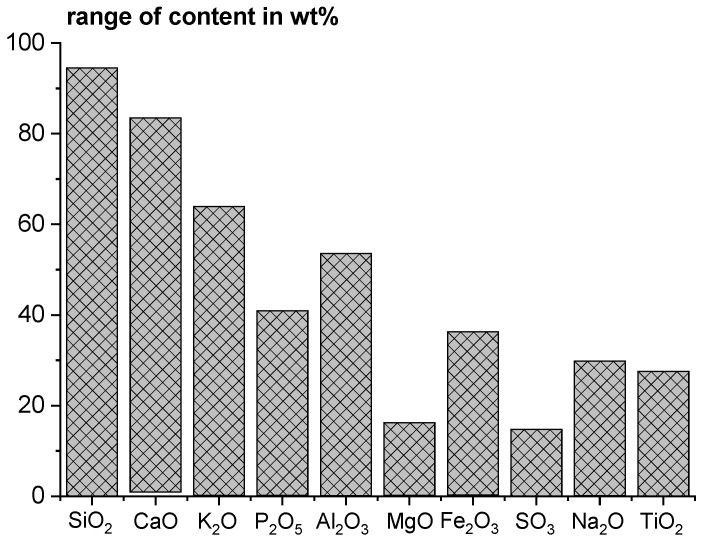
Ranges of chemical composition of a broad variety of biomasses (*n* = 86) based on high-temperature ash analyses (data from [4]).

At the same time, renewable energy is becoming increasingly important due to the climate targets. In this context, the proportion of energy generated from renewable fuels in Germany is increasing [3]. Around 65% of the final energy consumption of renewable energies for heat was generated by biogenic solid fuels in 2022 [3]. The combustion of biomass fuels is considered “CO_2_-neutral”, since only as much CO_2_ is released as had been absorbed during the growth phase. The ashes may be used as SCM, similar to the fossil fuel ashes used today. However, a major challenge when using ashes from plant-based and other biomass fuels is their diverse, variable composition. For example, ashes from different plants and different parts of plants differ considerably in their composition [4] (see Figure 1) and therefore also in their properties.

Depending on the growth location, the composition can likewise be affected by regional differences in nutrient availability. The composition and availability of biomass ashes also depend on the season. Furthermore, the pre-treatment, the burning conditions, the post-treatment (e.g., cooling procedure) and fractions (bottom ash, fly ash) influence the ash properties. Biomass is fed into the combustion chamber in different forms, for example, as wood chips, pellets, or bales of straw. There are various firing systems (e.g., pre-furnace firing, underfeed firing, grate firing, fluidized bed firing) whose suitability depends on the size of the plant and the nature of the fuel [5]. Also, in some cases there is a competing use of biomass ashes as fertilizer, if their composition is suitable. Further, many plants have a comparatively low throughput and are sparsely distributed. These circumstances make it difficult to establish biomass ashes as standardized SCMs. In addition, the substitution of clinker with biomass ash can impair the cement or concrete properties, e.g., water demand and workability, porosity and durability, as well as mechanical properties such as tensile and compressive strength [6]. Depending on the firing conditions, a high proportion of unburned carbon may be present which results in an extremely high specific surface area and a correspondingly high water demand. Other components can also have a negative effect, such as a high phosphate content which can inhibit hydration [7,8]. Some ashes also have very high potassium contents of up to more than 50 wt% [9] which can increase the pH in the cementitious system significantly.

In Germany, a distinction is made between stalk-type biomass and woody biomass, such as forest residues, the latter of which is the predominantly used plant-based solid fuel [5]. Stalk-type biomasses are, for example, residues from cereals or oil plants. Despite their generally good fuel value, their use as a fuel is more difficult due to their unfavorable combustion properties (elemental composition) compared to wood [10]. Although an extensive number of studies on plant-based biomass ashes for the application in the building materials industry do exist, the effects on cement and concrete properties have not been understood in detail yet due to the highly variable composition of the ashes.

Three wood ashes from running plants are investigated in this study. Although wood ash is considered only one type of biomass ash, the literature shows a range of CaO and SiO_2_ of about 1–63 and 9–79 wt%, respectively [11,12]. Studies show that the reactivity of wood ashes in terms of strength contribution is low or non-existent [11,12,13]. However, few studies also show increasing compressive strengths, mostly for low substitution levels of up to 10 wt% [14]. The ashes are ascribed pozzolanic and/or hydraulic properties [15,16].

Also, one spelt husk ash, as a stalk-type biomass ash, from a pilot-scale incineration was investigated in this study since the potential of the ash in small-scale experiments had already been shown by Putra et al. [17]. Spelt husk ash mainly consists of SiO_2_ and has a high potential for pozzolanic reactivity if appropriate combustion conditions are applied leading to a highly amorphous structure comparable to the well-researched rice husk ashes (compare [18,19,20]). As rice is not cultivated in Germany and other countries in temperate latitudes, the ash from spelt husks could be relevant instead.

Furthermore, one sample of a mineral residue from a lab-scale wet-chemical phosphorus recovery process from sewage sludge ash was investigated, the relevance of which will increase as described in the following. Phosphorus is an essential element for all living organisms, playing a vital role in energy metabolism and serving as a building block for DNA and RNA. Phosphate fertilizers used in agriculture contribute significantly to food security. Moreover, phosphorus is increasingly used in technical applications, such as LFP batteries for electromobility [21]. The primary phosphorus source is phosphate rock, whose limited reserves are mainly located and mined outside Europe in China, Morocco, the United States, and Russia. Consequently, the EU relies almost entirely on imports, leading the European Commission to classify phosphorus as a critical raw material [22]. Therefore, Switzerland and Germany require the recovery of phosphorus from sewage sludge and sewage sludge ash (SSA) from 2026 and 2029, respectively [23]. Also, other European countries are in the process of initiating similar directives. Compared to phosphorus recovery from sewage sludge directly, recovery from mono-incineration sewage sludge ash offers higher phosphorus concentrations, smaller material flows, and the highest recovery rates among commonly employed techniques, surpassing the legally required minimum recovery efficiency of 80 wt% [24]. In a wet-chemical process, phosphorus extraction from the ash matrix is achieved through the application of mineral acids, followed by the subsequent purification of the resulting acidic solution. Apart from the phosphorus product, about 50 wt% of the input ash remains as mineral residue (SSA-MR), for which a sustainable recycling strategy has yet to be devised, also to avoid landfilling costs. In terms of chemical composition, SSA-MR should in principle be suitable as SCM, as it is composed mainly of Si and Al and/or Fe, depending on the precipitation agent used in wastewater treatment. The limited potential of SSA as a component for building materials has already been investigated in the literature [25,26,27,28], however, some studies also report good pozzolanic properties [29,30]. Challenges such as the decrease in mechanical strength for samples with elevated ash content or the insertion of heavy metals into the building material when adding SSA need further investigation. In contrast, SSA-MR could have advantageous properties because of the applied treatment. Although the phosphate recovery is widely discussed in the scientific literature [31], only few studies exist on the use of SSA-MR as potential cement/concrete addition., e.g., Liang et al. [32] tested mortars with 15 wt% cement substitution by such mineral residues and found similar strengths as for the control mortar concluding that the mineral residues are suitable as a pozzolanic addition. This work therefore includes SSA-MR for first in-depth analyses.

This study characterizes the different biomass ashes and the residue from phosphate recovery concerning their physical, chemical, and mineralogical composition, including the analysis of trace elements and heavy metals in the solid and eluate. It aims to gain a better understanding of how composition affects performance. In the approach presented here, the influence on cement hydration was investigated with a combination of isothermal calorimetry and X-ray diffraction. The plausibility of the results was verified using thermodynamic modeling. The R^3^ reactivity [33], as well as the impact on mortar workability and strength were investigated to benchmark the performance. The pore structures of the hydrated systems were analyzed in order to link the interpretation of the hydration and strength results. Strength, cumulative heat, and clinker consumption were correlated to verify the overall plausibility of the study. The behavior of the materials in the cementitious system was qualitatively assigned to their sample characteristics as the widely varying compositions did not allow for statistical evaluation. The analysis of environmentally relevant parameters was included in this study in order to consider the potential of the different samples more holistically.

## 2. Materials and Methods

### 2.1. Materials

#### 2.1.1. Overview

Three wood ashes (WBA-DE, WFA-DK, WFA-UK) were investigated which originate from operating power plants burning untreated wood. WBA-DE originates from a power plant in Germany. The sample was provided in a slagged state (“bottom ash”) and had a moisture content of approx. 15 wt%, as it was cooled in a water bath after incineration. For this study, it was dried at 105 ± 5 °C to mass constancy and ground to approximately the target Blaine fineness of 5000 cm^2^/g in a laboratory ball mill. The other ashes were already delivered in a dry state. WFA-DK and WFA-UK were fly ashes from power plants in Denmark and the United Kingdom, respectively, which already had sufficient fineness. No further grinding or post-processing steps were carried out in order to consider the most realistic and economical utilization option. The spelt husk ash (SHA) originates from a pilot-scale plant (Ökotherm, co. A.P. Bioenergietechnik GmbH, Hirschau, Germany) at the German Biomass Research Center (DBFZ). The ash had a slag content of approx. 10 wt%. The slaggy part was also ground in the laboratory ball mill and mixed with the remaining fines. The SSA-MR was produced from sewage sludge ash that originates from the industrial-scale mono-incineration plant Bottrop (Emschergenossenschaft, Essen, Germany). At the Institute of Environmental Engineering (RWTH Aachen University) the SSA was treated in laboratory scale using 1.75 mol/L HCl with a mass ratio of HCl/SSA of 7.5 mixed in a glass beaker with a magnetic stirrer for 45 min. Then the resulting mineral residue was filtered using a Büchner funnel (co. Morgan Advanced Materials Haldenwanger GmbH, Waldkraiburg, Germany) followed by washing with demineralized water in a glass beaker with a magnetic stirrer until the pH was neutral. Afterwards the mineral residue was filtered again using a Büchner funnel. Table 1 summarizes the background information on the different ashes.

The cement used was a CEM I 42.5 R which meets the criteria of a test cement according to the fly ash standard DIN EN 450-1 [34]. Ground calcium carbonate (GCC) (ground marble) as a quasi-inert addition was used in several places for comparison with the performance of the ashes and the residue from phosphorus recovery.

#### 2.1.2. Chemical Composition

For the chemical analysis the samples were dried at 105 ± 5 °C to constant mass. The loss on ignition was determined according to EN 196-2 [35] at 950 °C using a muffle furnace. Chloride was determined using cold digestion according to EN 196-2 [35]. The determination of carbon and sulfur contents was conducted using a carbon/sulfur analyzer (Eltra CS-2000, co. ELTRA GmbH, Haan, Germany). Further elemental analysis was performed via X-ray fluorescence spectroscopy using a Panalytical Axios spectrometer (co. Malvern Panalytical GmbH, Kassel, Germany).

The results of the chemical characterization of the ashes are shown in Table 2 and of the reference cement and GCC in Table 3. SHA has a high carbon content, but contains no mineral carbonates (see Section 2.1.3). This indicates a high proportion of unburned organic matter. Also, the results for the WFAs indicate comparatively high contents of unburned organic matter. This can lead to a high water demand and reduce workability in the cementitious system. Also, different plant-related organic constituents are known to have strong negative impact on the cement hydration, such as saccharides and organic acids [36]. All ashes have comparatively high contents of potassium which can cause alkali-silica reaction with relevant aggregates [37]. Also, elevated alkali contents are known to accelerate early hydration by increasing the pore solution pH, which in turn enhances the dissolution of clinker phases and pozzolanic materials. However, a reduction in late-age strength is often observed at high alkali concentrations, which is attributed to the influence of alkalis on the morphology of the C-S-H phases [38,39,40]. SHA consists mainly of SiO_2_ (>60 wt%) and has a low CaO content (<3 wt%) and is therefore approximately comparable to rice husk ashes but with a higher alkali content [19]. For the wood ashes the Si/Ca ratio is more balanced. In terms of reactivity, a high CaO content can lead to (latent) hydraulic properties rather than pozzolanic if it is present in a reactive state [41]. Al_2_O_3_, as a further potential strength-forming component, makes up only a small proportion of all ashes (≤5 wt%). WFA-DK, WFA-UK and SHA have chloride contents >0.1 wt%, which can potentially have a corrosion-promoting effect when used in reinforced concrete and is currently not permitted, for example, for siliceous fly ash according to EN 450-1 [34]. WFA-DK shows the highest sulfate contents (~4 wt%). WBA-DE has low chloride and sulfate contents supposably because they were leached out during cooling in the water bath [42]. SSA-MR consists mainly of SiO_2_ (~56 wt%) and Fe_2_O_3_ (~20 wt%). The iron results from the flocculants used in the dewatering process of the sewage sludge, e.g., FeCl_3_ or Fe_2_(SO_4_)_3_ [43]. SSA-MR has low chloride and sulfate contents due to the leaching process. The P_2_O_3_ content is only 1.24 wt% from initial 12.53 wt% of the SSA showing that the phosphate leaching in the recovery process was efficient. Still, a considerable amount of unburned organic matter is present in the SSA-MR.

#### 2.1.3. Mineralogical Composition

Subsamples were manually ground with an agate mortar to a grain size below 63 µm. The mineralogical composition was then determined by X-ray diffraction using a Panalytical X’Pert Pro diffractometer equipped with an X’Celerator detector (co. Malvern Panalytical GmbH, Kassel, Germany). Data were measured in a range of 5 to 70 °2θ with a step size of 0.0167 °2θ and a total measurement time of 2 h. Rietveld refinement was performed to determine the quantitative composition of the samples using the software Panalytical Highscore Plus 4.8 (co. Malvern Panalytical GmbH, Kassel, Germany). The internal standard method with a rutile standard was used to quantify the amorphous content.

**Figure A1:**
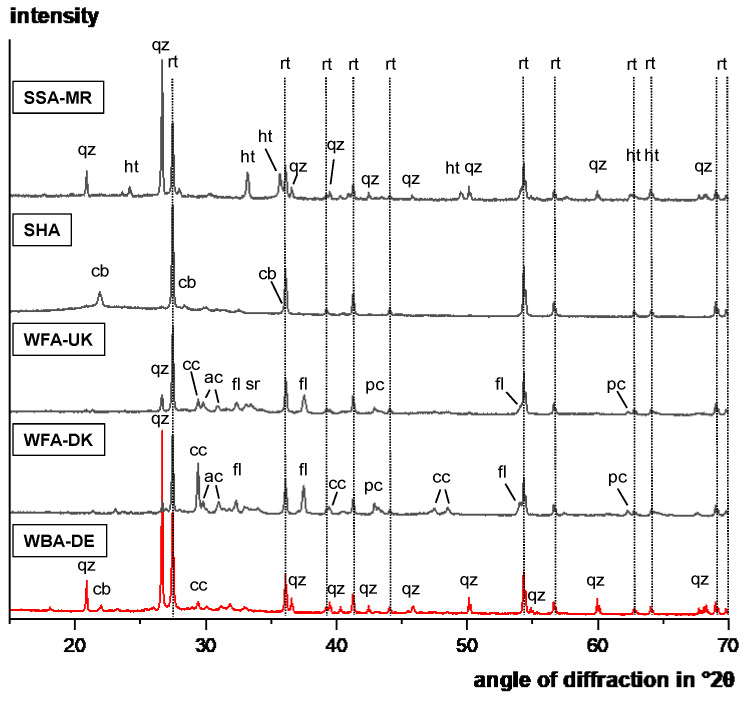
X-ray diffractograms of the ashes and the residue from SSA with identification of the main peaks (rt: rutile (internal standard); qz: quartz; ht: hematite; cb: cristobalite; cc: calcite; ac: arcanite; fl: free lime; sr: spurrite; pc: periclase).

The mineralogical composition of the materials is also shown in Table 2. Due to the complex mineralogy, the individual phases are mainly grouped together. The diffractograms of the ashes and SSA-MR are shown in Figure A1. It can be seen that the three wood ashes differ considerably in their composition. The wood fly ashes show significant amounts of free lime and periclase which is also found in the literature for wood ashes [15,44]. In contrast, WBA-DE has no periclase and free lime, as this bottom ash was cooled in water. However, it also contains significantly lower proportions of portlandite and carbonates. Possibly, free lime was partially dissolved during quenching in water. Remaining portions of the formed calcium hydroxide could have reacted with pozzolanically reactive components of the ash forming X-ray amorphous C-S-H phases during subsequent moist storage. This reaction is also suggested by Etiegni & Campbell [45]. In addition, the WBA-DE shows a higher proportion of silicates, whereas carbonates or easily carbonatable minerals (free lime and periclase) dominate as crystalline phases in WFA-DK and WFA-UK. High combustion temperatures favor the formation of free lime, whereas at lower combustion temperatures carbonates can also form directly during combustion [46]. In contrast to the WFA investigated by Ukrainczyk et al. [44], there are no clinker phases such as larnite or brownmillerite. The silicate phases are mainly quartz or cristobalite. The two fly ashes (WFA-DK and WFA-UK) show a similar composition due to the similar firing technology, whereby WFA-UK has a significantly higher amorphous content. This may be caused by the significantly higher firing temperatures (see Table 1). WBA-DE shows an X-ray amorphous share of about 55 wt%. The spelt husk ash SHA has a very high amorphous content of around 90 wt%, which initially indicates a metastable mineral phase and correspondingly high reactivity potential. However, the chemical data also indicate an increased proportion of unburned matter, which accordingly makes up a part of the amorphous phase determined. The mineralogical composition of SSA-MR is mainly quartz and hematite as well as an amorphous phase of about 40 wt%. Because of the amorphous share, all investigated samples show the potential for reactivity. However, it is well known that reactivity does not correlate with the amount of the amorphous share when comparing different materials [47,48].

#### 2.1.4. Physical Characterization

Laser granulometry was used to determine particle size distributions (Bettersizer S3, co. 3P Instruments GmbH & Co. KG, Odelzhausen, Germany), helium pyknometry was used to determine particle densities (AccuPyc 1330, co. Micromeritics GmbH, Unterschleißheim, Germany), and the 5-point BET method using nitrogen as adsorption gas was applied to determine the specific surfaces of the particles (a 3P sync 210, co. 3P Instruments GmbH & Co. KG, Odelzhausen, Germany). The Blaine value was determined in accordance with EN 196-6 [49].

**Figure 2:**
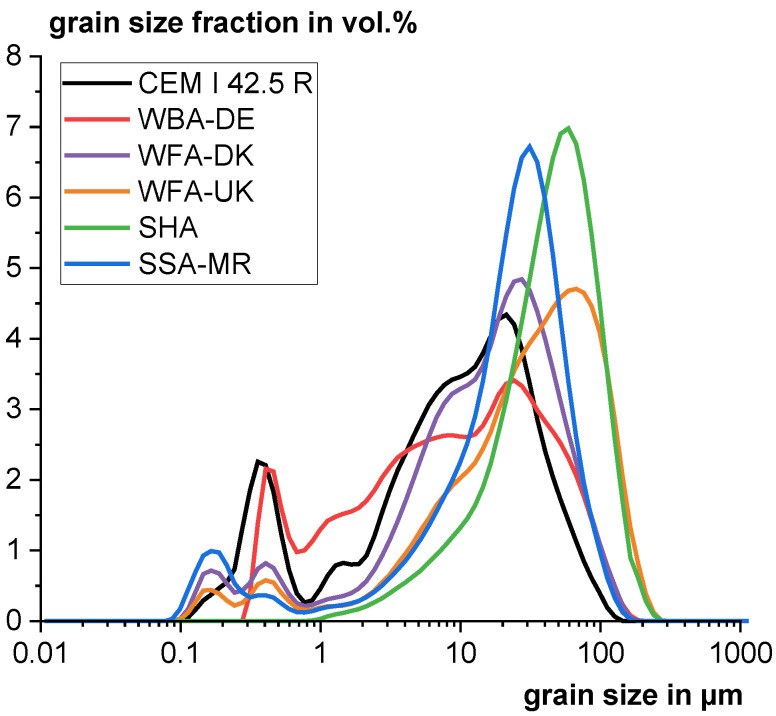
Grain size distribution of the ashes, the residue from sewage sludge ash and the reference cement.

The physical parameters investigated are also shown in Table 2. WBA-DE has the lowest specific surface area, as the material consists of ground bottom ash with low content of unburned organic matter. SHA has by far the highest specific surface area due to the large proportion of unburned organic matter. The high specific surface areas can lead to high water demand and poor workability in the cement system. The particle size range of all samples is within a suitable range for use as a cement addition. However, the particle size distribution of the high-carbon ashes WFA-UK and SHA are comparatively coarser (see Figure 2).

### 2.2. Methods

#### 2.2.1. Environmental Parameter

The solids content of trace elements and heavy metals was analyzed after digestion in aqua regia according to EN 13657 [50] using ICP-MS according to EN ISO 17294-2 [51]. Only mercury was measured using AAS according to EN ISO 12846 [52]. The determination of the PAK_16_ was performed according to EN 17503 [53] using gas chromatography. The determination of the PCB_6_ was performed according to EN 17322 [54] using gas chromatography and mass spectrometric detection.

The batch leaching test was performed according to EN 12457-4 [55] in double determination. 90 g of the sample and 900 g of deionized water were weighed into 1 L PE bottles. Then the samples were rotated for 24 h in an overhead rotator at 5 rpm. After removal, the samples were left to stand for 10 min to allow the solids to sediment. The eluates were then decanted and filtered through syringe filters with a 0.45 µm PES membrane. Also, a blank test was performed using deionized water without a sample to verify that no contaminations occurred. The pH was measured using a pH analyzer L40 (co. Mettler Toledo International Inc., Greifensee, Switzerland). The electrical conductivity was measured with an analyzer WTW LF 2000 (co. Xylem Analytics Germany Sales GmbH & Co. KG, Weilheim, Germany). Potassium, sodium and calcium were determined using a flame photometer (Elex 6361, co. Eppendorf SE, Hamburg, Germany), sulfate, chloride, phosphate and nitrate using ion chromatography (Dionix ICS 1100, co. Thermo Fisher Scientific Inc., Waltham, MA, USA). The trace elements and heavy metals were analyzed using ICP-MS according to EN ISO 17294-2 [51]. Only mercury was measured using AAS according to EN ISO 12846 [52].

#### 2.2.2. Grain Morphology

The particle morphology was investigated using scanning electron microscopy (SEM) (Gemini SEM 300, Co. Carl Zeiss AG, Oberkochen, Germany) on loose powders and polished sections of the powders embedded in epoxy resin. The samples were prepared with carbon coating to prevent charging. A backscattered and a secondary electron detector were used for visual analysis. Energy dispersive X-ray spectroscopy (EDX) was used to perform chemical analysis with the SEM.

#### 2.2.3. Reactivity Test

The reactivity of each sample was determined in accordance with ASTM C1897-20 [33] (also known as R^3^ test [56]) using an isothermal calorimeter (TAM Air, TA Instruments, New Castle, NY, USA). A potassium solution was prepared with 4.00 g of KOH and 20.0 g K_2_SO_4_ dissolved in 1.00 L of deionized water. The solid mix was prepared by shaking 15.00 g of calcium hydroxide, 2.5 g of calcium carbonate and 5.00 g of the sample in a closed container. All components were then tempered at 40 °C. A quantity of 27.00 g of the potassium solution was mixed with the solids for 2 min at 1600 rpm using a shear blender. Then two ampoules were immediately filled with 15 g of the paste each and placed in the calorimeter channels for a double determination. The measurement duration of the reaction heat was 7 days. The first 75 min were discarded in the evaluation in accordance with the standard.

#### 2.2.4. Hydration Study

##### Isothermal Calorimetry

The ashes, the leaching residue from sewage sludge ash and the GCC were investigated with isothermal calorimetry (TAM Air, TA instruments, New Castle, NY, USA) at 20 °C in the cement system as pastes using a w/b of 0.5 and 20 wt% cement substitution for each sample. After adding water to the solids, the blend was mixed at 1600 rpm for 2 min using a shear blender (EUROSTAR 60 digital, co. IKA-Werke GmbH & Co. KG, Staufen, Germany) in order to mimic concrete like mixing including shear stresses from the present aggregates. Subsequently, two ampoules were filled with 15 g of the paste each and placed in the calorimeter channels within 5 min after mixing for a double determination. The measurement duration of the reaction heat was 7 days.

##### X-Ray Diffractometry (XRD)

XRD hydration studies were conducted similar to the calorimetry studies on pastes with a w/b ratio of 0.5 using the pure CEM I 42.5 R as reference as well as blends with 20 wt% cement substitution using WBA-DE, WFA-DK, SHA and SSA-MR adapting the method described in [57]. The solids were weighed in and homogenized using a shear blender at 600 rpm for 2 min. After the addition of water, the cementitious pastes were blended for 3 min at 1600 rpm. Then, the pastes were filled in cylindric silicone molds with an inner diameter of 25 mm and 100 mm height. The formwork was carefully tapped on the table to deaerate and compact the samples without segregation. After sealing the molds with tape, the samples were cured in an overhead rotator at a rotation speed of 5 rpm to avoid segregation until being removed from the molds after 24 h. The top of the sample was cut-off, crushed and placed in the sample container. Deionized water was added for the storage of the rest of the sample. The crushed sample slice helps to prevent the rest of the sample from leaching while keeping the sample water-saturated. For the storage at 20 °C in the climate chamber, the sample container was sealed tightly by wrapping it with Parafilm and tape to prevent carbonation. A precision saw was used to cut two slices of approximately 2 mm at each measuring date at the age of 1, 2, 7, 28 and 90 days. Each first slice was discarded. The remaining slice was briefly rinsed with tap water to remove sawing residues and directly prepared for the XRD measurement. Immediately before the measurement, the slice was freshly polished in a few drops of deionized water on a silicon carbide abrasive paper. The fresh surface was then quickly rinsed with deionized water and immediately surface dried with a tissue. The mineralogical composition was then determined by X-ray diffraction using a Panalytical X’Pert Pro diffractometer equipped with an X’Celerator detector (co. Malvern Panalytical GmbH, Kassel, Germany). The data were measured in the range from 6 to 55 °2θ with a step size of 0.0334 °2θ and a total measuring time of 30 min. An external rutile standard was used for the determination of the X-ray amorphous share. Quantitative phase analysis was performed using the Rietveld refinement software Highscore Plus 4.8 (co. Malvern Panalytical GmbH, Kassel, Germany). Due to practical reasons only single measurements were performed, however, the plausibility of the results were validated by cross-comparison to literature results, thermodynamic modeling, cumulative heat and compressive strength results.

##### Thermodynamic Modeling

The software GEMS-PSI with the cemdata 18.1 database was used to model the hydration process [58]. As a first step, the phase composition obtained from the XRD analysis were used to fit the decrease in clinker phase contents over time with an allometric function. In the next step, based on the chemical and mineralogical data of the raw materials, an SHA and an SSA-MR phase were created in the program. The phase compositions of the reference blend and blends with a cement substitution level of 20 wt% with SHA and SSA-MR were entered into the program and normalized to 100 g of the total mixture. In reality, the C/S ratio of the C-S-H phase differs from the model in the program (C/S of 1.63 in the GEMS model [58] and C/S of 1.70–2.00 in real systems [59,60,61], however the ratio can be lower depending on the pozzolanic reaction). Therefore, the model was adjusted to a C/S ratio of 1.85 to transfer CaO from portlandite to C-S-H according to [47] (see Formula (1)).

(1) $$n(\mathrm{CaO}{)}_{\mathrm{corrected}}=\frac{(n(\mathrm{CaO})/n({\mathrm{SiO}}_{2}){)}_{\mathrm{corrected}}}{(n(\mathrm{CaO})/n({\mathrm{SiO}}_{2}){)}_{\mathrm{predicted}}}\;\cdot \;n(\mathrm{CaO}{)}_{\mathrm{predicted}}$$

The decrease in portlandite for each mix was calculated according to Formula (2).

n(Ca(OH)_2_) = (n(CaO)_predicted_ − n(CaO)_corrected_) · n(C-S-H) (2)

This calculation decreases the portlandite and increases the free water and C-S-H content in the model. Finally, since the consumption of X-ray amorphous SCM phases are difficult to track with XRD, the degree of SCM reaction was approached by adapting the modeled portlandite content to the experimental portlandite content measured by XRD.

#### 2.2.5. Pore Structure

The pore size distribution was analyzed using the mercury penetrometer AutoPore IV 9500 (co. Micromeritics GmbH, Unterschleißheim, Germany) with an incremental program with 41 pressure points in the range of 10 to 0.004 µm pore diameter. The pore size was calculated from the pressure using the Washburn equation assuming cylindric pores, a surface tension of mercury of 0.485 N/m and a contact angle between mercury and the pore wall of 140°. Pore diameters higher than 10 µm were considered as air voids and not included in the evaluation [62]. For preparation, the samples were cut from cement paste cylinders as slices at defined ages as described in Section 2.2.4. The slices were immersed in isopropanol in an air-tight vessel for solvent exchange. The isopropanol was renewed daily in the first four days. After 7 days, the samples were removed from the isopropanol and dried in a desiccator for 7 days at a low vacuum of 0.5 bar as it is assumed that a slight vacuum does not significantly alter the pore structure and prevents the sample from carbonation [57]. However, the 7-day storage in a low vacuum was longer than originally planned, as there was still a distinct odor of isopropanol during the first few days.

After drying, the samples were stored under argon atmosphere until measurement. The surface of the slices was abraded with sandpaper immediately before measurement to remove potentially carbonated layers. A double determination was applied.

#### 2.2.6. Setting Time, Soundness and Mortar Tests

The setting times were determined using the Vicat needle device according to EN 196-3 [63] using a cement substitution level of 25 wt% according to EN 450-1 [34].

The soundness was tested with the Le Chatelier ring according to EN 196-3 [63]. However, because of problems with determining the standard stiffness with 30 wt% cement substitution according to EN 450-1 [34] the mixtures were designed similar to the mortar samples with 20 wt% cement substitution and a w/b of 0.5.

The workability and compressive strength of mortars were investigated on blends with different cement substitution levels (10–25 wt%) using the different materials. The mortars were prepared with a mixture of water, binder and standard sand at a ratio of 0.5:1:3 according to EN 196-1 [64]. The prisms were stored for 24 h in the molds at a relative humidity of >95%. After demolding, the prisms were stored in a water bath at 20 ± 1 °C until strength testing. The compressive strength test comprises 6 replicas.

The mortar flows were determined using a Hägermann cone in accordance with EN 1015-3 [65]. They were adjusted as closely as possible to the reference without segregation with different amounts of superplasticizer (MasterGlenium ACE 460, Master Builders Solutions Deutschland GmbH, Trostberg, Germany) (see Table 4). When the superplasticizer addition exceeded 2 g its water content (~40 wt%) was subtracted from the water addition. The mortar flow was adjusted to the reference, since it was assumed that the differences in workability and consequently compactability have a significantly stronger impact on strength than differences in the contents of superplasticizer. The compressive strengths of the mortars were tested after 2, 7, 28, and 90 days.

## 3. Results and Discussion

### 3.1. Environmental Parameters

When using sustainable novel binders, environmental compatibility must be ensured. At first, the total solids content must be evaluated in order to prevent the accumulation of environmentally relevant parameters in the material cycle. Furthermore, the environmentally relevant parameters that can be mobilized must be investigated in order to assess the actual pollution potential. The assessment of the environmental impact of building materials is a complex topic for which there are various approaches. However, the aim here is not to make a full assessment, but rather to provide a general overview of the solids contents and eluate concentrations in a batch leaching test.

Table 5 shows the solids contents of inorganic and organic parameters of the different materials compared to literature data of up to 55 siliceous fly ashes from different power plants [66,67] and the German thresholds for the use of siliceous co-combustion fly ashes in concrete [68]. For the ashes, only cadmium of WFA-DK is significantly higher than the reference data for siliceous fly ashes and the German thresholds. SSA-MR shows significantly higher contents of barium, copper and selenium compared to the siliceous fly ashes [66]. The organic parameters PAK_16_ and PCB_6_ show very low concentrations for the ashes. They were not determined for SSA-MR as they were also assumed to be uncritical.

Table 6 shows data of the analyzed eluate parameters in the batch leaching test compared to literature data of siliceous fly ashes [66]. As German thresholds for siliceous fly ashes for the use in concrete do not exist for this test, for orientation the German thresholds for the landfilling of inert wastes are given [69]. PAK and PCB were not analyzed in the batch test, as no significant amounts were found in the solids. Beside a potential environmental impact of high concentrations of different substances, they may also impact the properties of the cementitious system as discussed in the following. For the wood fly ashes (WFA-DK and WFA-UK), the chromium release is significantly higher than the reference values and an environmental pollution can be expected in an unbound state. High chromium(VI) contents in the cementitious system are known to increase setting time and lower compressive strength [70,71,72]. For SSA-MR the selenium release is significantly higher than the reference data for siliceous fly ashes and may impact the environment in an unbound state. No major impact of selenium on strength is found in the literature. The sulfate content of WFA-DK is considerably higher than the reference data. Beside a possible environmental impact, high sulfate contents retard the initial hydration of C_3_S, and a more extended induction period is observed [73]. But, high sulfate contents can also cause false set after mixing or internal sulfate attack at later age. The nitrate release of WFA-DK and WFA-UK is comparatively high. Nitrate is known as accelerator for the cement hydration [74]. SHA shows a very high phosphate release which could delay hydration [7,8,29,75]. High potassium releases, such as for WFA-DK, WFA-UK and SHA can lead to alkali-silica reaction with reactive aggregates [37]. Also, it is known to enhance early hydration, however, to lower strength at later age [38,39,40]. WBA-DE shows no relevant release, as also found with other WBAs in the literature [15].

It must be emphasized here that leaching in deionized water in the batch test does not reflect the leaching from concrete. The eluate concentrations indicate some critical parameters for the environmental compatibility such as chromium and selenium. However, the actual environmental impact depends on the application case and should be evaluated in the hardened cement systems using, e.g., the dynamic surface leaching test [76]. It is well known from the literature that various substances are bound in the hydration products and prevented from leaching [77,78]. However, there are currently no uniform regulations and limit values in Europe for assessing the environmental compatibility of building materials. Therefore, consistent and standardized evaluation methodologies are required, such as source–path–target concepts, which integrate data from standardized leaching tests and modeling to describe leaching processes, the environmental distribution of substances, and their occurrence at relevant points of compliance [79].

### 3.2. Grain Morphology

**Figure 3:**
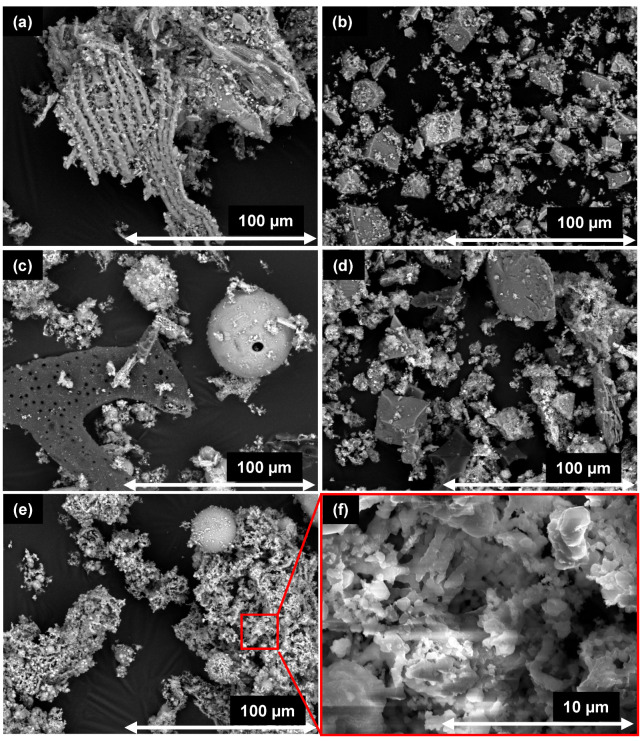
SEM-BSE images of (**a**) SHA, (**b**) WBA-DE, (**c**) WFA-UK, (**d**) SSA-MR, (**e**) WFA-DK and SEM-SE image of WFA-DK (**f**).

SEM was used to investigate the grain morphology and structure of the samples as possible contribution to the interpretation of their behavior in the cementitious system discussed in the following sections. It is generally known from literature that wood ash is irregularly shaped and porous [12]. Figure 3 shows the SEM figures of the grain morphology of the different materials. SHA shows the parallel presence of massive grains and elongated porous structures (see Figure 3a), which probably reflect the original morphology of the plant components. WBA-DE consists mainly of massive isometric grains, with a partly even surface (see Figure 3b). WFA-UK consists mainly of irregularly shaped particles of varying morphology (see Figure 3c). Spherical particles of different sizes (up to >100 µm) comparable to hard coal fly ash also occur. Comparably, Ukrainczyk et al. [44] also found spherically shaped wood fly ash particles occurring together with irregularly shaped ones. SSA-MR consists partly of massive isometric grains and partly of irregular shaped and porous particles (see Figure 3d). WFA-DK predominantly has finely structured grains with a filigree network and a porous structure (see Figure 3e,f). The EDX point spectra indicate, that these structures mainly consist of calcium carbonate and potassium sulfate, which is in line with the chemical and mineralogical analysis. Only a small proportion consists of spherical particles, which are rich in silicon and sometimes alumina. The variability of the ashes is accordingly also reflected in the grain morphologies. EDX measurements (not presented here) show very different chemical compositions at individual spots in all samples allowing no further conclusions within the frame of this study.

### 3.3. Reactivity Test

**Figure 4:**
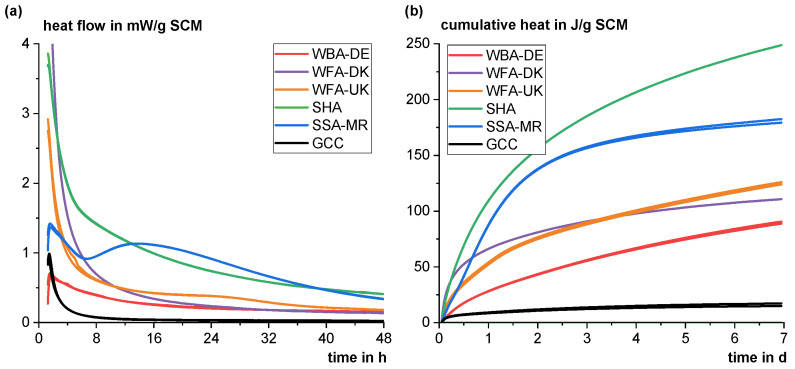
Heat flow up to 48 h (**a**) and cumulative heat up to 7 days (**b**) in double determination as a function of time of the different materials in the R^3^ test (ASTM C1897-20) using isothermal calorimetry.

Figure 4 shows the heat flow (a) and cumulative heat (b) of the different materials in the R^3^ test according to ASTM C1897-20. WFA-DK shows the highest heat flow at the beginning, which then decreases again most rapidly. The initial heat release is assumed to be due to the hydration energy released by free lime, which reacts immediately with water to form calcium hydroxide. The hydration of periclase is assumed to occur slower depending on grain surface morphology [80,81]. Even after 75 min (the first 75 min are neglected in the evaluation), reaction heat is still released from this process and bias the absolute value of the cumulative heat release with regard to pozzolanic (and/or latent hydraulic) reactions. The relatively rapid drop in the heat flow indicates that there is at most a very low pozzolanic (and/or latent hydraulic) reactivity. WFA-UK also contains free lime, so the heat development of this phase in the first few hours of the experiment also has an influence on the cumulative heat. However, the slightly higher heat release later on indicates a slightly higher pozzolanic (and/or latent hydraulic) reactivity. WBA-DE shows no or very low reactivity despite the relatively high amorphous content (~55 wt%). As discussed in Section 2.1.3, moisture and the presence of calcium hydroxide may have already caused potential pozzolanic components to react and form X-ray amorphous C-S-H phases. The SHA is the only ash showing a distinct reactivity comparable to common siliceous fly ashes [82]. The SSA-MR shows a distinct reaction hump beginning after about seven hours clearly indicating pozzolanic reactivity. The cumulative heat after 7 days of reaction is slightly lower than for common siliceous fly ashes and rather in the range of natural pozzolana according to [82]. Compared to a ground calcium carbonate (GCC), which is considered to be mostly inert, the wood ashes show a significantly higher heat release. However, as discussed above, this is not necessarily attributable to a pozzolanic (and/or latent hydraulic) reaction because of the complex chemical-mineralogical properties (see Section 2.1) and the overall rather low cumulative heat (compare [47,82]).

### 3.4. Hydration Study

#### 3.4.1. Isothermal Calorimetry

**Figure 5:**
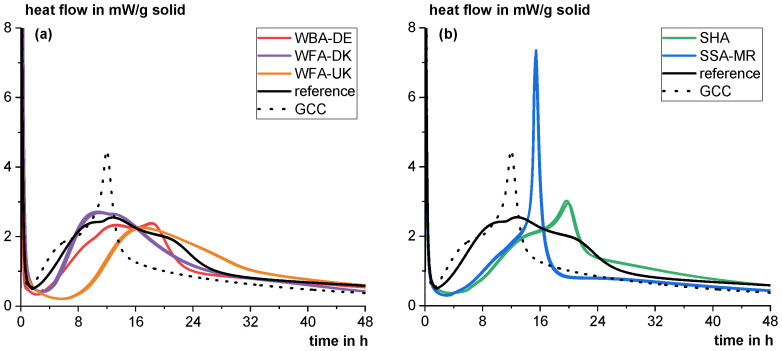
Heat flow in double determination as a function of time in the first 48 h of the hydration of cement pastes with a w/b ratio of 0.5 using CEM I 42.5 R including 20 wt% of the different materials using isothermal calorimetry (divided into two graphs (**a**,**b**) for clarity).

Figure 5 shows the heat flow from cement pastes with a cement substitution level of 20 wt%. The blend with WBA-DE shows a slight delay of the C_3_S hydration peak and the sulfate depletion peak is slightly visible close to the maximum of the C_3_S peak. WFA-DK leads to a comparable extension of the dormant period but then to an accelerated C_3_S reaction. The sulfate depletion is also significantly accelerated but still can be distinguished from the C_3_S peak. WFA-UK significantly alters the hydration of the cement. The C_3_S reaction starts with about 5 h delay and is then significantly decelerated. The extension of the dormant period for WFAs and lowering of the main peak was also observed, e.g., by Ukrainzyk et al. [44]. No sulfate depletion peak is visible for the WFA-UK blend. The SHA also significantly delays the C_3_S reaction to about 4 h but then show a C_3_S reaction peak whose maximum is fused together with the sulfate depletion peak. This intense peak is attributed to the second ettringite formation when, after depletion of the sulfates in the solution, the sulfate adsorbed on the C-S-H phases is desorbed leading to an intense reaction with C_3_A [83]. The early sulfate depletion inhibits the hydration of C_3_S [73,83,84], however, the reason for this is not yet clear [73]. The effect is comparable when using GCC and even more pronounced for the SSA-MR blend. The faster sulfate depletion with the use of SCM is attributed to a faster C-S-H formation due to the high specific surface area and hence a higher sulfate adsorption rate on C-S-H [83]. Here, a sulfate adjustment could help for a better performance in mechanical properties, especially in the early age. In contrast, the WFAs already provide higher sulfate contents (see Table 2). The reference GCC blend is the only one in which the dormant period is shortened compared to the reference with 100 wt% cement possibly due to the very high fineness (see Table 3).

**Figure 6:**
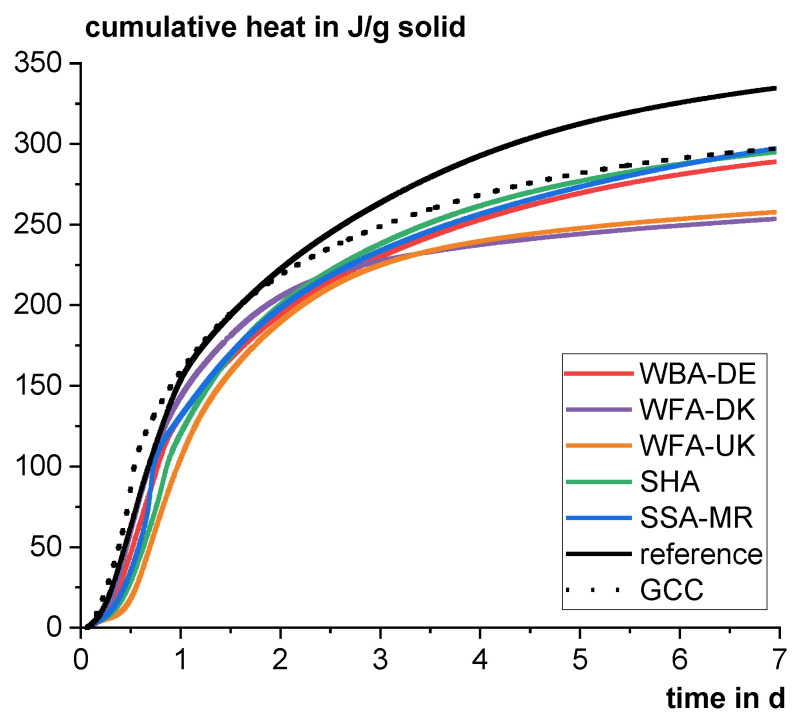
Averaged cumulative heat as a function of time of the hydration of cement pastes with a w/b ratio of 0.5 using CEM I 42.5 R including 20 wt% of the different materials using isothermal calorimetry.

In terms of cumulative heat after 7 days, all blends show lower hydration heat than the reference (see Figure 6). Concordantly, other studies have also found lower reaction heats compared to the reference using reactive biomass or coal fly ashes [85,86,87]. Several studies report a higher reaction heat when normalized to the cement fraction by using biomass ashes indicating an enhanced clinker reaction [29,87,88]. This does not apply here for the WFA blends at 7 days. The reaction rates are significantly lowered after about 3 days and are then significantly lower than for all other blends indicating an inhibition of the alite reaction.

#### 3.4.2. X-Ray Diffractometry

**Figure 7:**
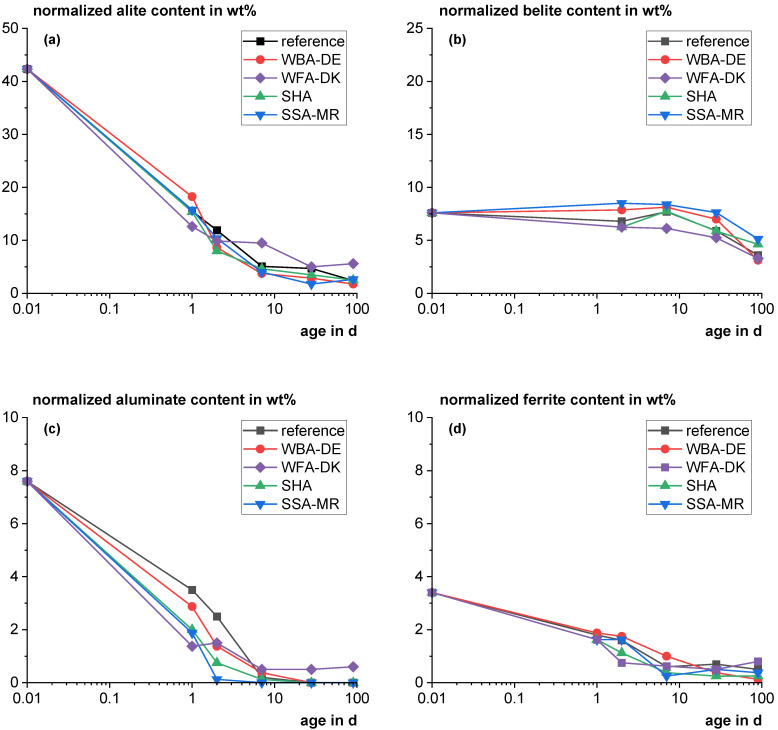
Clinker phase development as a function of time of the main phases (alite (**a**); belite (**b**); aluminate (**c**); ferrite (**d**)) for mixtures with 20 wt% cement substitution with the SCMs normalized to the cement proportion compared to a reference with 100 wt% cement. (For illustration in the logarithmic scale, the time point zero was set to 0.01 days.).

Figure 7 shows the clinker phase development as a function of time for mixtures with 20 wt% cement substitution with the SCMs normalized to the cement proportion compared to a reference with 100 wt% cement. WFA-UK was not included in the experiment. The WFA-DK blend shows the highest alite consumption after one day which is consistent with the high reaction rate observed in Section 3.4.1. However, after 2 days it reacts much slower than the other materials, which is also consistent with a low reaction rate observed in Section 3.4.1. For example, after 90 days, it has the significantly highest alite content, which is statistically supported by the Grubbs outlier test (G value: 1.74; critical vaue: 1.72; *p* value: 0.024). For the other blends and the reference, surprisingly, the overall course of alite consumption is quite similar and general differences cannot be identified within the precision of the method. The belite reaction is slow up to 90 days for all samples (comparable to [29,89]). The early aluminate (C_3_A) consumption is faster for the blends than for the reference. Similar findings were observed by Zunino & Scrivener for blended cements [83]. Comparable to the alite reaction, the WFA-DK shows the fastest aluminate (C_3_A) dissolution at one day and later the reaction is significantly inhibited. The aluminate clinker phase (C_3_A) could still be detected after 90 days for the WFA-DK blend. The ferrite reaction behaved similarly.

**Figure 8:**
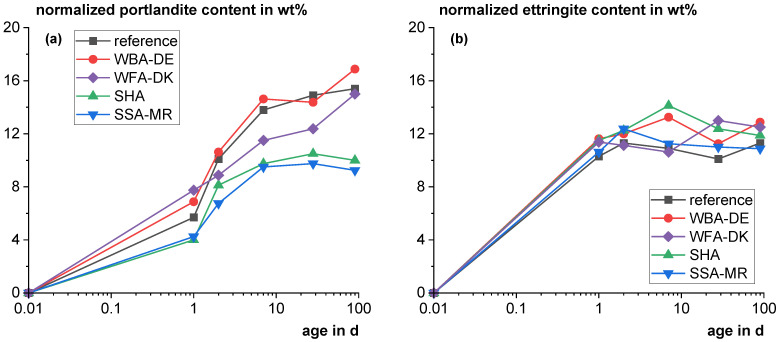

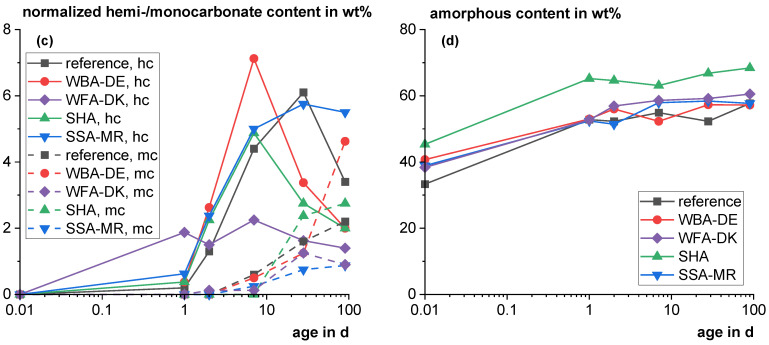
Development of the main hydrate phases (portlandite (**a**); ettringite (**b**); hemi-/monocarboaluminate (**c**) (hc: hemicarboaluminate; mc: monocarboaluminate); amorphous content (**d**)) as a function of time for mixtures with 20 wt% cement substitution with the SCMs normalized to the cement proportion (except the amorphous content) compared to a reference with 100 wt% cement. (For illustration in the logarithmic scale, the time point zero was set to 0.01 days.).

Figure 8 shows the development of the main hydrate phases as a function of time for mixtures with 20 wt% cement substitution with the SCMs normalized to the cement proportion (except the amorphous content) compared to a reference with 100 wt% cement. The portlandite contents revealed that the use of SHA and SSA-MR leads to a significant portlandite consumption and confirmed the pozzolanic reactivity found with the R^3^ test (see Section 3.3). The WBA-DE blend shows comparable portlandite contents to the reference confirming no significant reactivity. The WFA-DK blend shows a higher portlandite content on the first day, which results from the high initial free lime. Afterwards, the portlandite contents are slightly lower than for the reference but still rising up to 90 days. Surprisingly, the ettringite formation is quite similar for all blends., e.g., from the calorimetry curve it would be expected to be significantly higher at one day for the SSA-MR blend because of the strong secondary ettringite peak. The ettringite quantification seems to scatter comparatively high and, within the accuracy of the method, the ettringite content seems to stay relatively constant from 1 to 90 days for all blends. This is statistically supported by the *t*-test for regression coefficients, which showed for the various blends that the gradients of the linear regressions do not deviate significantly from zero (*p* values > 0.24). It is also supported by thermodynamic calculations in Section 3.4.3. The WFA-DK blend shows the highest hemicarboaluminate contents after one day but remains relatively constant afterwards, which is in line with the C_3_A reaction even though it has the highest calcite content (see Section 2.1.3). For all other blends and the reference, the hemicarboaluminate content significantly rises from the first day to day seven. Then it decreases because of its transition to monocarboaluminate, except for the mixture with SSA-MR. SSA-MR seems to stabilize the hemicarboaluminate phase more than the other materials. No iron-containing AFm phases were found for the blend with the iron-rich SSA-MR. When comparing chemical and mineralogical analysis of the SSA-MR (see Table 2) it becomes clear that most of the iron is incorporated in the crystalline hematite and therefore iron may have no significant impact on hydration. The amorphous content is more or less constant after the first day of the reaction. Only the blend with SHA shows significantly higher amorphous content than the other pastes due to the high amorphous content of SHA itself. No calcium monosulfoaluminate was detected for all mixtures as also found by Scrivener et al. [89] for Portland cement paste. This phase is often poorly crystalline and might contribute to the X-ray amorphous share. Also, the base cement contains small amounts of calcite which is known to stabilize ettringite with the formation of carbolauminate hydrates and suppress monosulfoaluminate formation [90,91].

Surprisingly, the general impact of the different materials (except WFA-DK) on the clinker hydration is relatively low. In contrast, Mejdi et al. [29] found a systematically lower reaction degree of the clinker phases when using SSA. Only WFA-DK seems to inhibit the hydration after a fast initial reaction which is in accordance with the calorimetry results. Traces of periclase were detected up to 28 days in the WFA-DK blend, however, no brucite could be found.

#### 3.4.3. Thermodynamic Modeling

**Figure 9:**
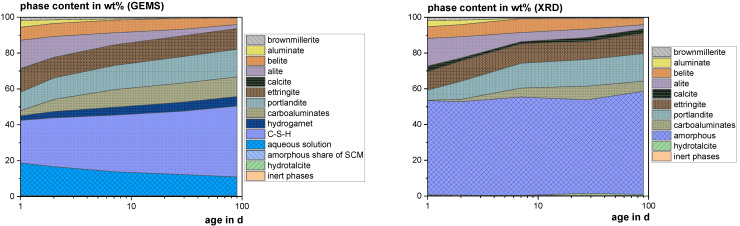
Modeled (GEMS) and measured (XRD) phase development of the reference mix using CEM I 42.5 R.

**Figure 10:**
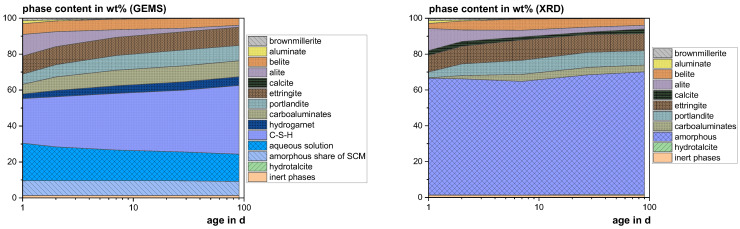
Modeled (GEMS) and measured (XRD) phase development of the mix with 20 wt% cement substitution using SHA.

**Figure 11:**
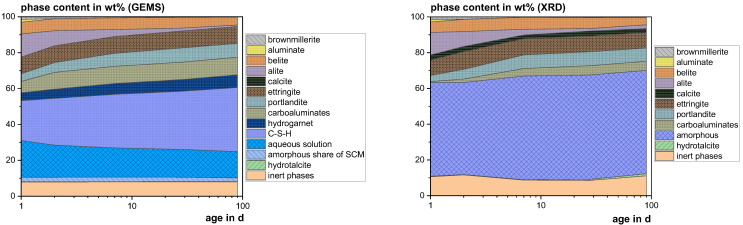
Modeled (GEMS) and measured (XRD) phase development of the mix with 20 wt% cement substitution using SSA-MR.

Figure 9, Figure 10 and Figure 11 show the experimental results (XRD) in comparison to the calculated ones (GEMS). The higher calcite contents in the XRD probably result from carbonation during the 30 min measurements. In the GEMS model, the X-ray amorphous share is represented by the C-S-H phase, the aqueous solution and the amorphous share of the SCM. Hydrogarnet can also be attributed to the amorphous share, as it was not found with XRD and generally occurs in low crystallinity [92]. The XRD results show significantly lower contents of carboaluminate hydrates, which are also known to occur partially X-ray amorphous due to their low crystallinity [93]. Considering all these phases, the model fits the XRD results quite well.

**Figure 12:**
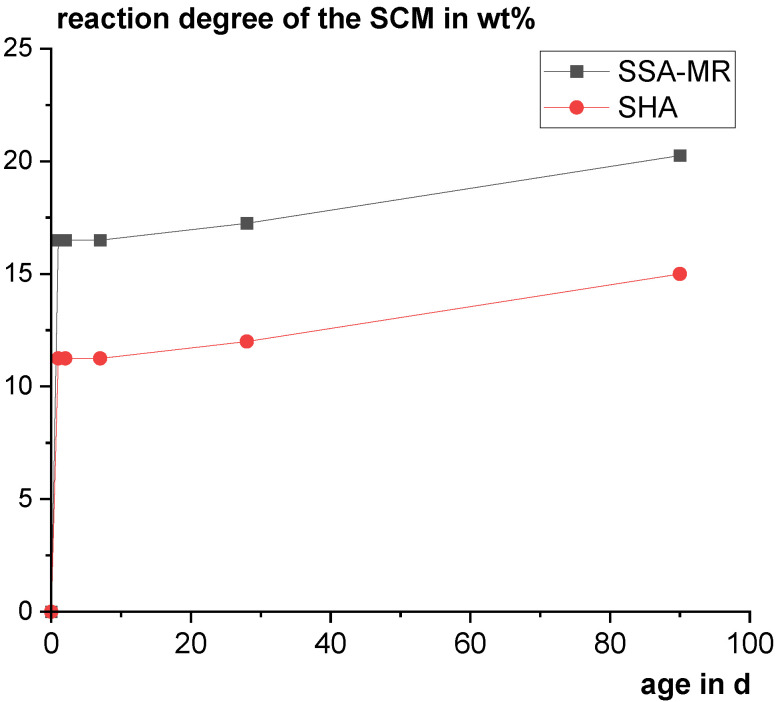
Reaction degrees of SHA and SSA-MR, estimated from the thermodynamic model based on the measured (XRD) portlandite content.

Considering the portlandite contents from XRD, relatively low reaction degrees of 15 wt% for SHA and 20 wt% for SSA-MR were estimated in the model (see Figure 12). The values are generally in a realistic range [94], however, the early age reaction degrees appear to be overestimated. Comparative tests are needed to validate the approach. Due to the relatively low reactivity of the samples, the carbonation during the measurement and the defined C/S ratio in the model (see Section 2.2.4) can significantly bias the results.

### 3.5. Pore Structure

The pore structure of the cementitious matrix in mortars or concretes is a decisive factor for the strength development as the effective cross-section on which the pressure is applied decreases with increasing proportion of pores. The pore structure is also a decisive factor for durability, as it determines the diffusion of substances into the building material. The term “pore entry diameter” is used here according to Scrivener et al. [57] instead of pore diameter, as mercury intrusion porosimetry only determines the size of the pore entry, which biases the results for bottle-neck pores. The limitations of the method are thoroughly discussed in [57].

**Figure 13:**
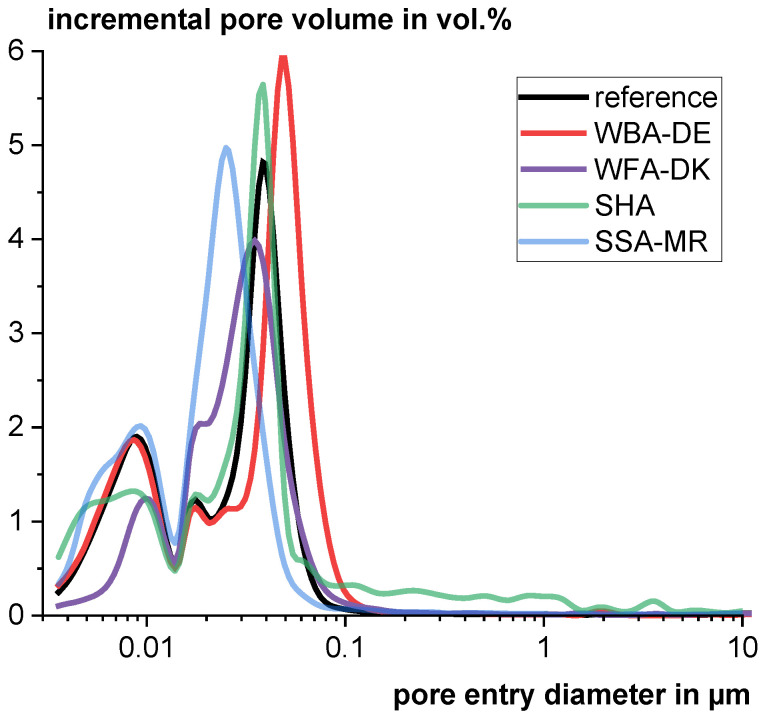
Averaged incremental pore volume as a function of the pore entry diameter of pastes with w/b ratio of 0.5 using CEM I 42.5 R including 20 wt% of different materials.

The use of the investigated materials leads to an increased total porosity for all samples (see Table 7). However, when comparing the pore size distribution (see Figure 13), the reactive SSA-MR leads to a significant pore entry refinement which is also known from literature for other reactive materials, such as calcined clays [95,96,97] or fly ashes [98,99]. The reactive SHA only leads to a slight refinement and also an increased porosity in the pore size range of 0.1 to 1 µm. This might be caused by the high content of unburned organic matter (see Section 2.1.2) and the pores of the residual structures as shown in Section 3.2. Surprisingly, also WFA-DK, which is considered to be unreactive and is assumed to have a negative impact on the hydration, leads to a slight pore entry refinement. Possibly, the porous particles (see Figure 3e,f) which have a much lower bulk density and make up a large volume proportion of the solids play a decisive role. It is conceivable that the porous particles absorb a huge proportion of the water, which significantly lowers the effective w/c ratio in the surrounding matrix. This could lead to a lack of water for clinker hydration and an early deceleration of hydration. The matrix around the porous ash particles could then be densified. However, the actual mechanism is not clear. When looking at the fine pore range, it becomes apparent that the gel pores and thus the C-S-H structure are poorly developed compared to the other blends. Only WBA-DE, considered as a nearly inert addition with a compact structure, showed higher total porosity and an enlargement of the pore structure, which is caused by the higher effective water/binder ratio.

### 3.6. Setting Time, Soundness and Mortar Tests

No standard stiffness according to EN 196-3 [63] could be determined for WFA-DK and WFA-UK, as these mixtures stiffen within a few minutes. The mechanism behind that is unclear but might be attributed to a high initial C-A-H formation if not enough sulfate was in solution in the first minutes. The XRD of the WFA-DK blend surprisingly showed that after one day some sulfate is still retained as arcanite phase and C_3_A has reacted most rapidly compared to the other blends. However, in situ XRD measurements after mixing would be needed for clarification.

The start of setting of a mixture with SHA with standard stiffness (w/b = 0.59) only occurred after around 11 to 13 h. This might be due to the high content of soluble phosphate and organic components. The start of setting of the WBA-DE blend with standard stiffness (w/b = 0.24) took place after 210 min and is therefore only slightly delayed compared to the reference (150 min) and comparable with conventional hard coal fly ash fulfilling the requirement according to EN 450-1 (shorter than twice the reference) [34]. Because of the problems with determining standard stiffness, for the determination of soundness for all mixtures a water/binder ratio of 0.5 was used with 20 wt% cement substitution. No significant expansion was observed, also not for the high free lime and periclase containing fly ashes (WBA-DE blend: 2.5 mm; WFA-DK blend: 4.5 mm; WFA-UK blend: 5.5 mm; SHA: 4.5 mm). Due to the limited sample amount available of SSA-MR, the setting time and soundness were not tested.

**Figure 14:**
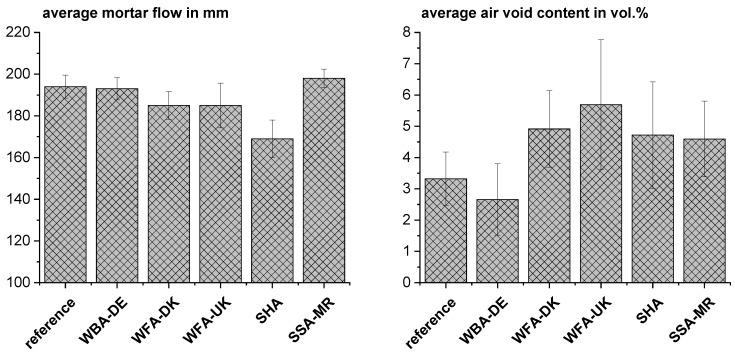
Average mortar flow and calculated average air void content with the test series using the different materials.

Mortar tests with different levels of cement substitution (10–25 wt%) were performed using the different materials. Figure 14 shows the average mortar flow and bulk density of the test series using the different materials. For overview, mortars with different levels of substitution were considered together as an average for each material. It was attempted to adapt the mortar flows to the reference in order to achieve a comparable compaction of the mortars (see Table 4). Only with SHA it was not feasible to achieve a much higher mortar flow than 180 mm without considerable segregation. The mortars with WBA-DE were the only ones where no superplasticizer was needed as WBA-DE consists mainly of compact isometric grains with even surface as shown in Section 3.2. The water demand is similar as for the reference cement (not different according to the *t*-test at a significance level of 0.05 (t value: −0.65; *p* value: 0.52)). The air void contents of all mortars calculated from the true densities of the individual components and the bulk densities of the mortar bars lie below 6 vol.% in average. The mortar containing WBA-DE shows very good compaction as it also showed good workability. According to the *t*-test, the average air void contents of the reference mortars and the WBA-DE mortars do not differ from each other at a significance level of 0.05 (t value: −1.08; *p* value: 0.29). Whereas for the other blends, the air void contents are significantly higher according to the ANOVA test at a significance level of 0.05 (F value: 7.4; *p* value < 0.001) by about 2–3 vol.%. Higher air contents in mortars blended with wood ash were also found, e.g., by Carevic et al. [15]. Therefore, a slight effect on the compressive strength can be expected.

**Figure 15:**
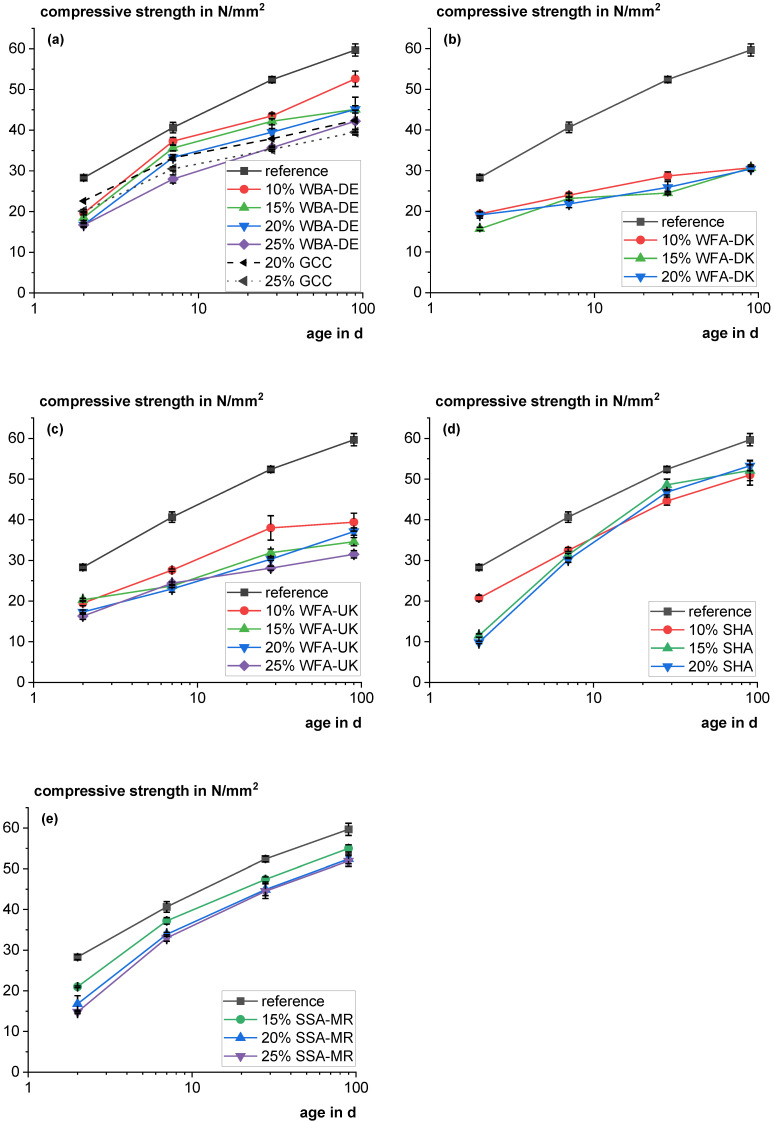
Mortar strength using the different materials (WBA-DE (**a**); WFA-DK (**b**); WFA-UK (**c**); SHA (**d**); SSA-MR (**e**)) for different cement substitution level compared to a reference with 100 wt% cement; (**a**) also includes references with ground calcium carbonate (GCC) for comparison.

Figure 15 shows the compressive strengths of the different mortar series compared to the reference with 100% cement in the binder fraction. Figure 15a additionally contains a reference with 20 and 25 wt% of ground calcium carbonate (GCC), as the previous results suggest that the ash acts as a filler. It becomes visible that substituting cement by WBA-DE significantly lowers the strength. Compared to the GCC blend it shows lower early age strength. However, contrary to the GCC mortar it still shows a significant strength increase between 28 and 90 days and already catches up at 28 days (not different according to the *t*-test with a significance level of 0.5 for substitution levels of 20 wt% (t value: 2.03; *p* value: 0.084) and for 25 wt% (t value: 1.53; *p* value: 0.16)). After 90 days, the strength of the WBA-DE mixture is slightly higher than the strength of the GCC mixture. The difference is statistically significant at a significance level of 0.05 according to the *t*-test for the substitution level of 20 wt% (t value: 4.91; *p* value < 0.001) and is just above the significance threshold for the substitution level of 25 wt% (t value: 2.37; *p* value of 0.059). Therefore, WBA-DE can be ascribed a filler function or very slight reactivity. The higher strength of the GCC in the first period might be caused by a better filler effect due to the high fineness or the enhanced carboaluminate hydrate formation when using extra GCC [47]. As WBA-DE was only ground in a laboratory ball mill, its potential as filler might be optimized with the adjustment of grinding. WFA-DK and WFA-UK showed a negative impact on the mortar strength. As discussed in the previous sections for WFA-DK, the hydration is inhibited after a few days leading to a lower formation of strength-building C-S-H phases. Also, the effect might be attributed to the low bulk density of the ash particles, as a large proportion by volume of the binder fraction consists of weak, porous particles. As there is a constant slow increase in strength over time, other destructive effects, such as expansion due to delayed MgO hydration, are improbable. However, it does not explain why there are no major differences in strength at different substitution levels, e.g., the compressive strength after 90 days is not different according to the ANOVA test with a significance level of 0.05 (F value: 0.29; *p* value: 0.75).

The SHA shows significant strength contribution. The course of the graphs indicates, that up to 10 wt% substitution the reaction kinetic is quite similar to that of Portland cement but with lower strength. At higher substitution levels the early age strength is significantly altered, e.g., by the high soluble phosphate or organic content as discussed in previous sections, but the late age strength is then improved by a higher pozzolanic reaction. Also, the SSA-MR shows a significant strength contribution with relative compressive strengths at a substitution level of 25 wt% of 0.85 after 28 days and 0.87 after 90 days.

**Figure 16:**
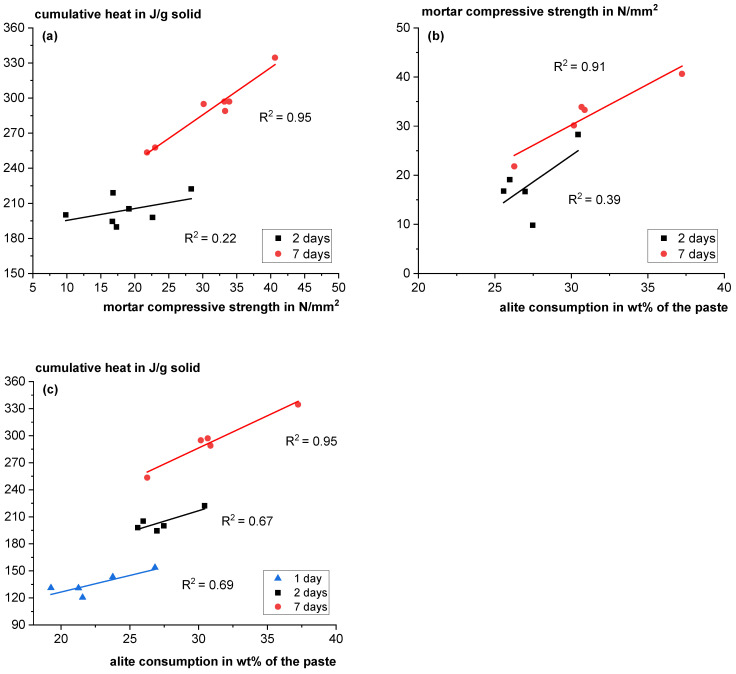
Correlation between cumulative heat and mortar compressive strength (**a**), mortar compressive strength and alite consumption (**b**) and cumulative heat and alite consumption (**c**) at different ages of the pastes and mortars.

Mortar compressive strength, cumulative heat from the calorimetry investigations (see Section 3.4.1) and alite consumption from the XRD investigations (see Section 3.4.2) are compared to check the overall plausibility of the results. When the mortar compressive strength is plotted against the cumulative heat, a good correlation is revealed (see Figure 16a). Comparable is found in the literature [100,101]. However, it appears reasonable that physical effects, such as differences in air void contents and packing density, impact strength development independently from the reaction heat. The correlation becomes better at later age. Similar findings were observed for the alite consumption and the compressive strength at 7 days (see Figure 16b). Frolich et al. [100] stated that the development of strength is more complex as it is not only a function of how much the clinker phases have reacted, but also depends on how the hydrated phases are deposited and connected, which might contribute here to the deviations, especially at early age. According to Jansen et al. [102], the heat release during Portland cement hydration can directly be ascribed to the hydration heat of the clinker phases, especially C_3_S and C_3_A. Also, surprisingly good correlations were found between alite consumption and the heat of reaction for the different blends (see Figure 16c).

## 4. Conclusions

− WFA-DK seems to significantly alter the C-S-H formation since it shows a major negative impact on mortar compressive strength. Whereas the 2-day-strength was comparable with the other blends, no considerable strength increase was observed up to 90 days. Using calorimetry and XRD, it was found that after a rapid initial reaction of the clinker, the reaction rate becomes very slow after a few days. After 28 days, only comparatively small amounts of gel pores had formed, confirming the inhibition of C-S-H formation. The reason for this is not yet clear and might be caused by different overlying negative effects, such as a high concentration of alkalis, soluble chromium and organic components as well as the irregular shaped grain morphology with a highly porous network. A similar strength development was found for WFA-UK. The calorimetry curve indicates a similar behavior; however, due to the low performance, WFA-UK was not investigated more closely.
− WBA-DE shows low contents of chloride and sulfate and no considerable reactivity but perfect workability because of a low content of unburned organic matter and mainly dense isometric particles with even surface. Therefore, it is suitable to be used as filler in cement or concrete. However, the grinding could be adjusted to improve early age performance.
− SHA shows good reactivity, but bad workability in a blended cement due to a very high content of unburned organic matter. Also, the setting time is delayed and the early age strength is low which might be attributed to a high content of soluble phosphate and organic components. As this ash originates from a pilot plant, the burning condition should be adjusted.
− SSA-MR shows a strength contribution comparable to SHA; however, less superplasticizer is needed and the early age strength is slightly better. However, the release of selenium in a batch leaching test appears to be critical from an environmental perspective.

Table 8 shows a comparative summary of the properties of the different samples with a qualitative evaluation. Due to the multitude of factors that influence reactivity and strength development in cementitious systems, which may counteract or superimpose one another, and the high variety and complexity of the composition of the biomass ash-based materials, a clear interpretation of their behavior becomes exceedingly difficult. Although different samples show a basic applicability, for a widespread use, quality control by the adjustment of burning conditions and fuel compositions is suggested.

## Figures and Tables

**Table 1:** Background information on the samples.

Designation	Country of Origin	Fuel	Firing System	Fraction	Burning Temperature
WBA-DE	Germany	wood chips	grate firing	bottom ash	~650 °C
WFA-DK	Denmark	wood pellets	dust firing	fly ash	~900–1000 °C
WFA-UK	United Kingdom	wood pellets	dust firing	fly ash	~1400–1500 °C
SHA	Germany	spelt husk ash	pilot plant, fixed bed furnace	total ash fraction	~750 °C
SSA-MR *	Germany	sewage sludge	stationary fluidized bed combustion	fly ash	>850 °C

* wet chemical phosphorus recovery process applied after incineration.

**Table 2:** Data of the chemical, mineralogical and physical characterization of the ashes and the residue from sewage sludge ash.

Parameter	Unit	WBA-DE	WFA-DK	WFA-UK	SHA	SSA-MR
LOI	wt%	3.62	16.49	14.48	16.13	8.22
total sulfur as SO_3_		0.13	4.12	1.88	0.72	0.42
total carbon as C		0.78	4.27	8.55	9.96	4.65
chloride		<0.01	0.37	0.19	0.26	<0.01
Na_2_O		0.01	0.01	0.62	0.01	0.81
K_2_O		5.61	10.36	7.11	10.65	1.63
Na_2_O equivalent		3.70	6.82	5.30	7.02	1.88
MgO		2.23	6.45	4.73	1.28	0.95
Al_2_O_3_		2.61	1.67	5.16	0.29	7.80
SiO_2_		60.62	12.19	28.65	62.73	55.98
P_2_O_5_		2.65	3.83	2.40	3.35	1.24
CaO		19.95	38.33	28.05	2.65	0.53
TiO_2_		0.17	0.11	0.52	0.02	1.19
MnO		0.43	1.29	1.54	0.06	0.08
Fe_2_O_3_		1.47	1.65	3.65	0.22	20.10
periclase	wt%	n.d.	4.6	2.3	n.d.	n.d.
free lime		n.d.	11.1	7.1	n.d.	n.d.
portlandite		0.5	1.7	0.6	n.d.	n.d.
carbonates		3.9	27.6	12.4	n.d.	n.d.
sulfates		0.4	9.5	7.4	3.0	1.4
phosphates		1.5	2.5	0.3	n.d.	1.7
chlorides		n.d.	0.3	0.3	0.3	n.d.
silicates		38.4	5.1	3.6	6.5	41.4
iron oxides		n.d.	n.d.	n.d.	n.d.	13.9
X-ray amorphous		55.4	37.7	66.3	90.1	41.7
true density	g/cm^3^	2.67	2.82	2.59	2.17	2.75
Blaine value	cm^2^/g	4960	9930	4502	4294	7537
BET surface area		21,450	24,790	140,000	419,000	271,000
D_10_	µm	0.7	1.6	3.6	10.3	2.3
D_50_		9.6	16.0	32.1	42.6	23.0
D_90_		51.6	49.4	102	96.9	55.1

n.d.: not detected.

**Table 3:** Data of the chemical, mineralogical and physical characterization of the reference materials.

Phase	Unit	CEM I 42.5 R	GCC
LOI	wt%	2.25	-
total sulfur as SO_3_		3.26	
total carbon as CO_2_		1.87	43.50
chloride		0.02	-
unsoluble, HCl/KOH		1.07	
Na_2_O		0.2	0.07
K_2_O		0.78	0.07
Na_2_O equivalent		0.71	-
MgO		1.55	1.50
Al_2_O_3_		5.55	0.21
SiO_2_		21.21	0.95
P_2_O_5_		0.11	-
CaO		62.75	53.60
TiO_2_		0.31	-
MnO		0.07	
Fe_2_O_3_		2.43	0.10
alite	wt%	63.5	-
belite		11.4	
aluminate (C_3_A)		11.4	
brownmillerite		5.1	
arcanite		-	
anhydrite		2.1	
bassanite		1.6	
gypsum		2.4	
calcite		1.7	95.8
dolomite		-	3.7
quartz		0.7	0.5
true density	g/cm^3^	3.14	2.73
Blaine value	cm^2^/g	3200	13,500
BET surface		10,870	35,010
d_10_	µm	0.4	0.4
d_50_		9.4	2.1
d_90_		36.8	6.6

**Table 4:** Superplasticizer addition and averaged mortar flow of the different mixtures.

Sample	Cement Substitution Level	Superplasticizer	Mortar Flow
	wt%	wt% from Binder	mm
reference	0	0	194
WBA-DE	10	0	197
	15		194
	20		193
	25		189
WFA-DK	10	0.3	186
	15	0.6	188
	20	0.7	180
WFA-UK	10	0.6	181
	15	0.9	178
	20	1.3	189
	25	1.4	193
SHA	10	0.4	172
	15	0.9	164
	20	2	173
SSA-MR	15	0.4	203
	20	0.4	200
	25	0.6	196

**Table 5:** Content of different environmental parameters in the solids compared to data of siliceous fly ashes (SFA) from [66,67] and German thresholds for the use of siliceous fly ashes generated with co-combustion in concrete [68].

Parameter	Unit	WBA-DE	WFA-DK	WFA-UK	SHA	SSA-MR	SFA [66,67]	Reference Threshold [68]
antimony	mg/kg	<1	1	4	<1	14	1.2–101	-
arsenic		1.1	2.4	5.0	<0.8	10.5	5–321	150
barium		318	215	262	66	4530	475–1645	-
lead		19	21	11	<2	127	11.4–817	700
cadmium		0.7	13.3	6.3	<0.2	0.6	0.403–7.0	10
chromium		25	57	72	3	139	29.4–355	600
cobalt		3	8	9	<0.1	21	9.7–126	-
copper		44	151	84	19	461	21–312	400
molybdenum		<2	5	5	3	22	7.15–189	-
nickel		8	14	26	5	166	26–298	500
mercury		<0.07	<0.07	0.08	<0.07	2.86	0.027–1.3	5
selenium		<1	<1	<1	<1	55	1.5–25	-
thallium		<0.2	1.4	0.5	<0.2	1.3	0.1–3.0	7
vanadium		10	8	28	< 1	58	54.8–1764	1500
zinc		77	2280	1010	116	2150	53–1200	1500
PAK_16_		<0.026	<0.016	<0.037	<0.039	n.a.	<0.001	30
PCB_6_		<0.0006	<0.0006	<0.0006	<0.0006	n.a.	0.0005–0.0008	0.5

**Table 6:** Eluate parameter from the batch leaching test according to EN 12457-4 compared to literature data of siliceous fly ashes (SFA) [66] and German thresholds for the landfilling of inert waste [69].

Parameter	Unit	WBA-DE	WFA-DK	WFA-UK	SHA	SSA-MR	SFA [66]	Reference Threshold [69]
pH	-	12.40	13.01	12.89	10.89	4.95	10.66–12.90	5.5–13
el. conductivity	µS/cm	6770	45,150	26,235	7085	300	1190–3180	-
antimony	µg/L	<1	<1	<1	<1	8.80	<7–22.7	6
arsenic		<1	<1	<1	11	11.7	3.9–29.2	50
barium		518	419	534	142	159	336–900	2000
lead		18	3	<1	<1	<1	<1.4	50
cadmium		<0.2	<0.2	<0.2	<0.2	<1	<0.2	4
chromium		34	2520	1690	25.5	<1	50.7–421	50
cobalt		<0.2	<0.2	<0.2	0.3	<1	<0.8	-
copper		7	<1	<1	4	4.99	<1.3	200
molybdenum		11	450	251	183	38.6	452–1660	50
nickel		<1	<1	<1	6	7.45	<0.9	40
mercury		<0.1	<0.1	<0.1	<0.1	<0.1	<0.5	1
selenium		<1	53	40	<1	625	10–278	10
thallium		<0.2	<0.2	<0.2	<0.2	<1	0.9–2.3	-
vanadium		<2	<2	<2	9	2.93	41.3–283	-
zinc		3	107	54	125	48.7	<1.3	400
sodium	mg/L	24.2	200	390	15.1	19.5	93.5–308	-
potassium		450	10,400	4510	2860	13.0	60.3–102	-
calcium		340	400	365	3.4	1.9	517–1235	-
chloride		3.40	345	208	210.5	10.2	<0.1–20.9	1500
sulfate		21.5	2590	984	544	35.5	351–766	2000
fluoride		0.1	0.3	<0.1	<0.1	-	-	1
nitrate		0.3	745	154	0.5	-	-	-
phosphate		<7.5	<7.5	<7.5	557.4	-	-	-

**Table 7:** Total porosity of paste samples considering pores < 10 µm using MIP.

Sample	Total Porosity
	vol.%
reference	25.5
WBA-DE	29.4
WFA-DK	27.1
SHA	30.4
SSA-MR	31.4

**Table 8:** Summary of the properties of the different samples with qualitative evaluation.

Property	WBA-DE	WFA-DK	WFA-UK	SHA	SSA-MR
environmental compatibility	neutral	negative		neutral	negative
R^3^ reactivity	neutral			positive	
effect on clinker hydration	neutral	negative		neutral	
strength contribution	neutral	negative		positive	

## Data Availability

The original contributions presented in this study are included in the article. Further inquiries can be directed to the corresponding author.
